# Beyond language: empathy and emotion recognition deficits in primary progressive aphasias

**DOI:** 10.1016/j.nicl.2025.103852

**Published:** 2025-07-23

**Authors:** Giulia Giacomucci, Alice Pieri, Valentina Moschini, Chiara Crucitti, Sonia Padiglioni, Carmen Morinelli, Giulia Galdo, Filippo Emiliani, Matilde Nerattini, Silvia Bagnoli, Assunta Ingannato, Sandro Sorbi, Benedetta Nacmias, Valentina Berti, Valentina Bessi

**Affiliations:** a Department of Neuroscience, Psychology, Drug Research and Child Health, University of Florence, Florence, Italy; b University of Florence, Florence, Italy; c Research and Innovation Centre for Dementia-CRIDEM, AOU Careggi, Italy; d SOD Neurologia I, Dipartimento Neuromuscolo-Scheletrico e degli Organi di Senso, AOU Careggi, Florence, Italy; e Regional Referral Centre for Relational Criticalities - Tuscany Region, Italy; f IRCCS Fondazione Don Carlo Gnocchi, Florence, Italy; g Department of Biomedical, Experimental and Clinical Sciences “Mario Serio”, University of Florence, Florence, Italy; h Nuclear Medicine Unit, Azienda Ospedaliero-Universitaria Careggi, Florence, Italy

## Abstract

Although primary progressive aphasia (PPA) is considered a language disorder, increasing evidence points to the presence of social cognition impairments in PPA variants. The aims of this study were to explore empathy and emotion recognition deficits in the three PPA variants (sv-PPA, lv-PPA, nfv-PPA) and to identify their neural correlates.

Eleven sv-PPA, 34 lv-PPA,11 nfv-PPA patients and 34 healthy controls (HC) were included in this study. Empathy was explored with the Interpersonal Reactivity Index (IRI) (Perspective Taking – PT, Fantasy – FT, Empathic Concern – EC, Personal Distress – PD), rated by caregivers before (T0) and after (T1) the onset of cognitive symptoms. Emotion recognition was evaluated with the Ekman 60Faces (EK-60F) Test and metabolic activity with [18F]FDG-PET.

In all PPA variants, PT score was reduced from T0 to T1 (sv-PPA *p* = 0.014, lv-PPA *p* < 0.001, nfv-PPA *p* = 0.022) and PD score was increased (sv-PPA *p* = 0.033, lv-PPA *p* < 0.001, nfv-PPA *p* = 0.009). Only lv-PPA showed a decrease of FT score (*p* = 0.024), while EC was spared in all three variants. Sv-PPA patients had the worst performances in the EK-60F Test, followed by lv-PPA and, lastly, by nfv-PPA.

Correlations between EK-60F scores and metabolic activity were found in sv-PPA and lv-PPA, highlighting the involvement of areas participating in the emotion recognition network: cingulate cortex, insula, temporal and orbitofrontal cortices and inferior frontal gyrus.

All PPA variants exhibited impairments in cognitive empathy (PT) and heightened emotional contagion (PD). The most severe deficits in emotion recognition were shown by sv-PPA, while nfv-PPA was the less impaired variant.

## 1 Introduction

Primary progressive aphasia (PPA) is a group of neurodegenerative syndromes characterized by a progressive and relatively isolated language impairment (Mesulam et al., 2021). On the basis of language deficits, distribution of brain atrophy (or hypometabolism) in imaging studies, and underlying pathogenic mechanism, three variants of PPA can be described: a semantic variant (sv-PPA), a non-fluent variant (nfv-PPA), and a logopenic variant (lv-PPA) (Gorno-Tempini et al., 2011). Sv-PPA and nfv-PPA are considered as language manifestations of the Fronto-temporal Lobar Degeneration (FTLD) spectrum, while lv-PPA is generally described as an atypical variant of Alzheimer's Disease (AD) (Ruksenaite et al., 2021, Spinelli et al., 2017). Although language impairments represent the main clinical manifestation of PPA syndromes (Mesulam et al., 2021), it is increasingly studied whether these deficits remain isolated or if they are accompanied by a deterioration of other cognitive functions, particularly of social cognition and empathy (Adolphs, 2009, Arshad et al., 2020, Fittipaldi et al., 2019).

Empathy is a complex construct, generally identified as the ability to share and understand another’s emotions and feelings (Decety and Jackson, 2004). Several models aiming to describe its functioning have been proposed – the most accepted being that of Decety and Jackson (Decety and Jackson, 2004) – and they all agree on the presence of two basic components of empathy: a cognitive and an affective one (Shamay-Tsoory, 2011, Fischer et al., 2019). Cognitive empathy consists of high level cognitive processes and allows one to infer and understand the others’ feelings and motivations, adopting their point of view, while affective empathy is a simpler and phylogenetically older function, which guarantees the automatic sharing of the other’s emotions (Shamay-Tsoory, 2011). One of the main components of affective empathy is emotional contagion, a primitive empathic response which represents a core function of empathy, necessary for all higher-level (both affective and cognitive) empathic processes (Shamay-Tsoory, 2011, Fischer et al., 2019, de Waal and Preston, 2017, Preston et al., 2002). Studies concerning social cognition impairments in PPA variants are scarce, but they generally point to the presence of at least some empathy deficits in these syndromes (Arshad et al., 2020, Fittipaldi et al., 2019).

In particular, deficits in both cognitive and affective empathy and in emotion recognition have been observed in sv-PPA patients (Arshad et al., 2020, Fittipaldi et al., 2019, Younes et al., 2022). Evidence concerning nfv-PPA is less consistent: cognitive empathy is generally described as impaired (Fittipaldi et al., 2019, Hazelton et al., 2017), similarly to emotion recognition capacity (Kumfor et al., 2013, Couto et al., 2013), while affective empathy seems to be spared or only slightly impaired (Fittipaldi et al., 2019, Hazelton et al., 2017). Finally, very few studies have investigated social cognition deficits in lv-PPA and have found a sparing of affective empathy, with an increase of emotional contagion (Fittipaldi et al., 2019, Hazelton et al., 2017, Giacomucci et al., 2023), and a reduction of cognitive empathy (Hazelton et al., 2017, Giacomucci et al., 2023). Results regarding emotion recognition impairments in this variant are inconclusive (Fittipaldi et al., 2019), as this capacity has been described both as preserved (Piguet et al., 2015) or impaired (Hazelton et al., 2017, Giacomucci et al., 2023). Overall, deficits in this latter function, if present, are referred to as milder than in the other two variants (Hazelton et al., 2017).

With this study with an exploratory nature, we aimed to confirm the presence of empathy and emotion recognition deficits not only in sv-PPA, but also in lv-PPA and nfv-PPA, and to better define differences among variants, evaluating the severity and extent of such impairments, and exploring their neural correlates using [18F]FDG-PET. Indeed, considering the previous works regarding social cognition deficits in PPA variants, we hypothesized that lv-PPA, sv-PPA and nfv-PPA patients might show different degrees of empathy and emotion recognition impairments. In particular, we expected sv-PPA patients to exhibit greater deficits than the other two variants, as sv-PPA has been described as the most similar to the behavioural variant of Fronto-Temporal Dementia (bv-FTD) (Van Langenhove et al., 2016). We also expected to find some differences between nfv-PPA and lv-PPA, which have rarely been compared due to their different underlying pathologies (FTLD and AD). Given that nfv-PPA falls within the FTLD spectrum, it might be associated with greater empathy deficits compared to lv-PPA. Moreover, considering that the three PPA variants are characterized by the involvement of distinct brain regions, we hypothesized that the differences in emotion recognition among the PPA variants could be associated with a loss of function in specific brain areas involved in the neural networks for emotion recognition. Thus, we hypothesized to find, for each PPA variant, specific correlations between hypometabolism at [18F]FDG-PET and emotion recognition impairments, preferring this technique to others (for example MRI) because it detects pathological changes before structural degeneration occurs, with higher sensitivity and specificity (Belder et al., 2024, Kiymaz et al., 2025, Routier et al., 2018).

## 2 Methods

### 2.1 Participants

We longitudinally included patients referred to our centre for language disturbance fulfilled the current consensus criteria for PP (Mesulam et al., 2021). Patients were classified into PPA variants by three neurologists (GG, SM and VB) and three neuropsychologists (VM, CM and SP) with expertise in cognitive disorders, according to the current diagnostic criteria of PPA (Gorno-Tempini et al., 2011). Prior established exclusion criteria were: i) non-native Italian speakers; ii) patients with a history of head injury, current neurological and/or systemic disease, or substance use disorder; iii) right predominant sv-PPA; and iv) patients with severe language impairment that did not allow neuropsychological evaluation. In particular, 12 nfv-PPA, 18 sv-PPA and 9 lv-PPA patients were excluded because of severe language impairment. Ultimately, 56 patients were included in the study: 11 nfv-PPA, 11 sv-PPA and 34 lv-PPA.

All participants underwent family and clinical history collection, neurological examination, extensive neuropsychological investigation, including speech and language testing, and evaluation of empathy through Interpersonal Reactivity Index (IRI) (Davis, 1983, Albiero et al., 2007) and facial emotion recognition capacity through Ekman 60Faces (EK-60F) Test (Ekman and Friesen, 1976, Dodich et al., 2014). A positive family history was defined as one or more first-degree relatives with documented cognitive decline. Age at empathy assessment was defined as age at IRI and EK-60F tests administration. Age at onset was defined as age at the onset of cognitive symptoms. Disease duration as the timeframe from the onset of symptoms and empathy evaluation. The onset of cognitive symptoms was determined as the moment patients or their caregivers reported the first language impairments to have appeared.

All the patients underwent brain magnetic resonance imaging (MRI) or computed tomography (CT) and [18F]FDG-PET. Eleven nfv-PPA, 10 sv-PPA and 32 lv-PPA patients underwent lumbar puncture for CSF collection, with analysis of biomarker (Aβ1-42, Aβ1-42/1–40 ratio, t-tau, p-tau). Three patients (1 sv-PPA and 2 lv-PPA) who refused lumbar puncture underwent amyloid-PET. Fourteen patients (1 nfv-PPA, 3 sv-PPA and 10 lv-PPA) underwent both CSF biomarkers analysis and amyloid-PET (Supplementary Table 1).

**Table 1:** Demographic features in non-fluent variant (nfv-PPA), semantic variant (sv-PPA) and logopenic variant (lv-PPA) Primary Progressive Aphasia patients and healthy controls (HC).

	HC	nfv-PPA	sv-PPA	lv-PPA
	n = 34	n = 11	n = 11	n = 34
*Sex (F/M)*	15/19	6/5	4/7	17/17
*Age at onset (years)*	−	67.83 ± 5.21	61.90 ± 9.55	65.67 ± 7.08
*Age at empathy evaluation (years)*	65.38 ± 11.36	71.47 ± 6.10	67.07 ± 8.09	69.48 ± 6.37
*Disease duration (years)*	−	4.09 ± 3.47	3.72 ± 1.67	3.55 ± 1.69
*Family history of AD*	17/34 (50.00 %)	4/12 (33.33 %)	8/11 (72.72 %)	18/34 (52.94 %)
*Years of education*	13.41 ± 11.36	12.41 ± 5.10	12.27 ± 3.79	12.55 ± 4.19

Values are reported as mean and standard deviation or frequencies or percentages for continuous variables and categorical variables respectively. M: males; F: females.

Methods used for CSF collection and analysis, brain FDG-PET and amyloid-PET acquisition and rating are described in further detail elsewhere are described in further detail elsewhere (Bessi et al., 2021, Giacomucci et al., 2021, Giacomucci et al., 2023, Minoshima et al., 1997).

PPA patients were compared to 34 healthy controls (HC), who underwent family and clinical history collection, global screening cognitive evaluation (Mini-Mental State Examination, MMSE), evaluation of empathy through IRI and facial emotion recognition capacity through EK-60F Test. In order to be included, potential HC needed a normal score at MMSE (adjusted for age, sex, and education based on normative data) confirming the absence of cognitive impairments.

Study procedures and data analysis were performed in accordance with the Declaration of Helsinki and with the ethical standards of the Committee on Human Experimentation of our Institute. The study was approved by the local Institutional Review Board (reference 15691oss). All individuals involved in this research agreed to participate and agreed to have details and results of the research about them published.

### 2.2 Neuropsychological assessment

All PPA patients were evaluated by an extensive neuropsychological battery consisting of global measurements (MMSE) and specific tasks exploring each cognitive function. Verbal and spatial short-working and long-term memory were explored through Digit and Visuo-spatial Span forward and backward (Monaco et al., 2013), Rey auditory Verbal Learning test immediate recall RVLT-I and delayed recall RVLT-D (Carlesimo et al., 1996), Babcock Short Story Immediate and Delayed Recall (De Renzi et al., 1977), and Rey-Osterrieth complex Figure recall (Caffarra et al., 2002). Attention was explored using Trail Making Test A (Giovagnoli et al., 1996) and visual search (Della Sala et al., 1992). Category Fluency Task (Novelli et al., 1970), Phonemic Fluency Test (Spinnler and Tognoni, 1987), and the Screening for Aphasia in NeuroDegeneration battery (Catricalà et al., 2017) were used to evaluate language. Constructional praxis was evaluated by Rey-Osterrieth Complex Figure copy (Caffarra et al., 2002), while executive functions by Trail Making Test B (Giovagnoli et al., 1996) and Stroop Test (Caffarra et al., 2002).

### 2.3 Interpersonal Reactivity Index (IRI)

Empathy deficits were assessed using the Interpersonal Reactivity Index (IRI) (Davis, 1983, Albiero et al., 2007, Maddaluno et al., 2022), a tool designed to measure empathic sensitivity by capturing both cognitive and affective components. The IRI comprises a 28-item questionnaire, divided into four distinct 7-item subscales, each addressing a specific facet of empathy:

• Perspective Taking (PT): Assesses the ability to adopt another person’s viewpoint.
• Fantasy (FT): Evaluates the tendency to identify with fictional characters.
• Empathic Concern (EC): Measures the inclination to feel compassion, concern, and warmth towards others experiencing distress.
• Personal Distress (PD): Gauges general anxiety and emotional reactions to uncomfortable situations.

The PT and FT subscales primarily reflect cognitive empathy, while the EC and PD subscales are indicative of the affective domain. Notably, PT and EC subscales are frequently utilized as indices of empathy by patients’ caregivers (Eslinger et al., 2011, Shamay-Tsoory et al., 2009)*.* Conversely, the PD subscale has been employed to measure emotional contagion, characterized by an automatic, complete identification with another’s behavior, facilitating affective incentives and altruistic actions (Decety and Jackson, 2004, Sturm et al., 2013). Each item on the IRI is presented as a statement, and respondents indicate their level of agreement on a 5-point Likert scale, ranging from 1 (does not describe me/the patient at all) to 5 (describes me/the patient very well). Some items are negatively worded relative to the overall sense of the subscale, requiring score inversion before analysis. Lower FT, PT and EC scores indicate a reduction of empathy capacity, while higher PD scores indicate a pathological increase of emotional contagion.

IRI was rated by informants, since caregivers’ ratings of empathy turned out to be an effective way for evaluation of patients affected by dementia (Rankin et al., 2006). Informants were asked to rate patients’ empathy at the present moment (T1) – after the onset of cognitive symptoms – and to give a retrospective evaluation of patients’ empathy before the onset of cognitive symptoms (T0). On the other hand, HC self-rated their empathy only at T1.

### 2.4 Ekman-60 faces test

Facial emotion recognition was assessed by Ekman-60Faces (EK-60F) Test, which consists in 60 black and white pictures of the Ekman and Friesen series of Pictures of Facial Affect (Ekman and Friesen, 1976), representing the faces of ten actors (six women and four men), each showing one of the six basic emotions (anger, sadness, happiness, fear, disgust, surprise). A global score (EK-60F global score) of 60 indicates the best possible performance. Each basic emotion has a sub-score of maximum of 10 points. Images were shown each for 5 s according to the Ekman and Friesen procedure (Ekman and Friesen, 1976), via power point presentation on a computer. Patients were asked to indicate which of the six basic emotions better represented the facial emotion shown on the display. Patients’ performance in recognizing facial emotions was classified as normal or pathological based on specific cut-offs proposed for the standardization of the test in the Italian population (Dodich et al., 2014).

### 2.5 Statistical analysis

All statistical analysis were performed via IBM SPSS Statistics Software Version 25 (SPSS Inc., Chicago, USA) and Jamovi Software version 2.3.21.0. All p-values were two-tailed and significance level for all analyses was set at α = 5 %, corresponding to a threshold *p* of 0.05. All variables were described as mean and standard deviation. Distribution of all variables was assessed through Shapiro-Wilk test. Depending on the distribution of our data, we used t- tests or non-parametric Mann-Whitney-U tests for between-groups comparisons and Pearson’s r or Spearman’s ρ for correlations. We used chi-square tests to compare categorical data. Differences among groups in continuous variables were assessed through one-way ANOVA followed by Bonferroni *post-hoc* test. We used analysis of covariance (ANCOVA) including age at empathy evaluation as a possible confounding factor. Differences between T0 and T1 scores were explored through Wilcoxon signed-rank test. We calculated the size effect by the Cohen’s d, r, partial η^2^, and Cramer’s V.

### 2.6 SPM analysis

In order to assess the metabolic patterns related to emotion recognition ability in the 3 PPA variants, we included patients who had undergone an (Piguet et al., 2015)FDG-PET scan within a temporal range of 18 months before or after the empathy analysis. (Piguet et al., 2015)FDG PET data were analyzed using Statistical Parametric Mapping (SPM12) on MATLAB (MathWorks Inc, Sherborn, MA, USA). Scans were manually reoriented, setting the origin to the anterior commissure, normalized to dementia-specific [18F]-FDG-PET template, and then smoothed (FWHM 8 mm). Correlation analyses were performed using multiple regression design, with age and MMSE as nuisance variables. The significance threshold was set at *p* < 0.001, uncorrected, and *p* < 0.05 FWE small volume corrected. Only clusters containing more than 20 voxels were deemed to be significant.

### 2.7 Data availability and open practices

Readers seeking access to the data should contact the corresponding author Dr. Valentina Bessi (valentina.bessi@unifi.it). Access will be granted to named individuals in accordance with ethical procedures governing the reuse of sensitive data. Specifically, requestors must complete a formal data sharing agreement. No analysis code was used. No part of the study procedures or analyses were pre-registered prior to the research being conducted.

## 3 Results

### 3.1 Description of the sample and comparison among PPA variants

Demographic variables are described in Table 1. Out of 56 PPA patients, 11 were diagnosed with nfv-PPA (19.64 %), 11 with sv-PPA (19.64 %) and 34 with lv-PPA (60.71 %). PPA patients did not differ in age at empathy evaluation, age at onset of cognitive disturbs, disease duration, years of education, MMSE and sex. Out of 54 patients who underwent *APOE* genotype analysis, 31.48 % were classified as *APOE* ε4 carriers. PPA patients were compared to 34 HC matched for age, sex and years of education.

### 3.2 Changes of empathy capacity along time

We compared IRI scores at T0 and T1 to determine whether there were statistically significant changes in IRI scores before and after the onset of cognitive symptoms in the three PPA variant subgroups (Table 2). A significant increase of PD scores was found in nfv-PPA (Wilcoxon’s W 0.00, *p* = 0.009, r = -1.00), sv-PPA (Wilcoxon’s W 1.00, *p* = 0.033, r = -0.999) and lv-PPA (Wilcoxon’s W 0.00, *p* < 0.001, r = -1.00). On the contrary, PT scores decreased from T0 to T1 in all PPA variants (nfv-PPA Wilcoxon’s W 28.00, *p* = 0.022, r = 1.00; sv-PPA Wilcoxon’s W 36.00, *p* = 0.014, r = 1.00; lv-PPA Wilcoxon’s W 295.00, *p <* 0.001, r = 0.967). Only lv-PPA showed a decrease in FT scores from T0 to T1 (Wilcoxon’s W 151.50, *p* = 0.024, r = 0.595). We also observed a reduction of EC scores from T0 to T1 in sv-PPA, but such decrease did not reach statistical significance.

**Table 2:** Comparison of IRI T0 and T1 scores in non-fluent variant (nfv-PPA), semantic variant (sv-PPA) and logopenic variant (lv-PPA) Primary Progressive Aphasia patients.

	nfv-PPA			sv-PPA			lv-PPA		
	Media ± SD	*W*	*p*	Media ± SD	*W*	*p*	Media ± SD	*W*	*p*
FT 0	18.54 ± 3.20	13.00	0.733	17.77 ± 4.59	31.50	0.313	18.00 ± 5.56	151.50	**0.024**
FT 1	17.27 ± 3.71			15.77 ± 5.84			16.42 ± 5.96		
PT 0 PT 1	21.63 ± 2.90	28.00	**0.022**	19.55 ± 6.59	36.00	**0.014**	23.09 ± 5.76	295.00	**<0.001**
PT 0 PT 1	18.00 ± 5.13			14.44 ± 7.36			17.60 ± 6.61		
EC 0	26.81 ± 4.23	10.00	0.098	25.00 ± 4.15	20.00	0.058	26.48 ± 4.16	154.00	0.066
EC 1	24.63 ± 4.96			22.22 ± 6.55			24.72 ± 6.11		
PD 0	17.27 ± 3.82			17.33 ± 5.70			17.24 ± 5.91		
PD 1	23.36 ± 4.38	0.00	**0.009**	22.77 ± 5.44	1.00	**0.033**	25.93 ± 7.00	0.00	**<0.001**

Values are reported as mean and standard deviation. Statistically significantly different values between the groups are reported as **bold character**. Statistical significancy at *p* < 0.05. FT Fantasy; PT Perspective Taking; EC Empathic Concern; PD Personal Distress.

### 3.3 Evaluation of facial emotion recognition ability in PPA patients and HC

**Fig. 1:**
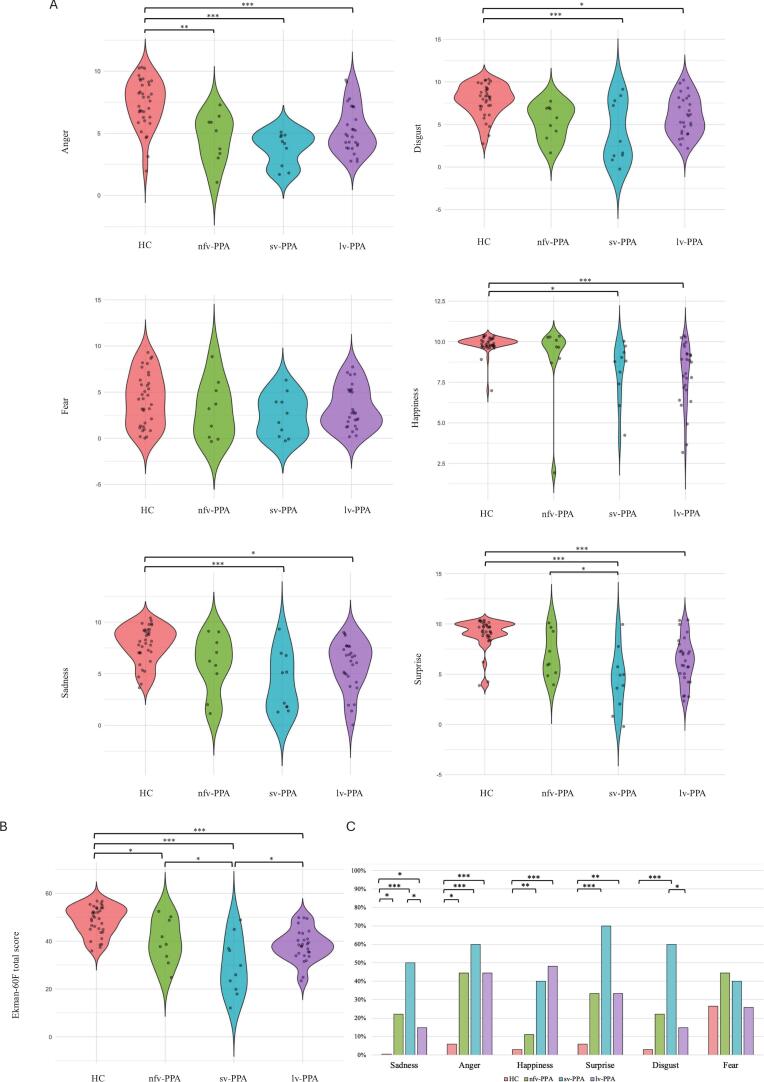
Emotion recognition assessed by Ekman-60 Faces test (EK-60F) in non-fluent, semantic and logopenic Primary Progressive Aphasia (nfv-PPA, sv-PPA, lv-PPA) and healthy controls. A) Differences in single emotion recognition scores. Values quoted in the y-axis indicate single emotion recognition scores. Horizontal bars indicate significant differences between groups. B) Differences in Ekman-60 Faces total score (corrected for age and educational level). Values quoted in the y-axis indicate Ekman-60 Faces total scores. Horizontal bars indicate significant differences between groups C) Percentage of patients with pathological scores in single emotion recognition. * p < 0.05; **p < 0.01; ***p < 0.001.

Facial emotion recognition ability was assessed by EK-60F test and was compared among the three PPA variants (Supplementary Table 2; Fig. 1). EK-60F total scores (adjusted for age and years of education) were significantly different between HC and the three PPA subgroups (F [3, 76] = 19.831, *p* < 0.001, partial η^2^ 0.439). In more details, sv-PPA patients showed the worst performance, with lower scores (29.58 ± 11.98) than lv-PPA (38.37 ± 6.67, *p* = 0.015, Cohen’s d −1.16), than nfv-PPA (39.89 ± 9.34, *p* = 0.024, Cohen’s d 1.36), and than HC (48.58 ± 6.03, *p* < 0.001, Cohen’s d 2.51). Both nfv-PPA and lv-PPA performed poorer than HC (nfv-PPA vs HC *p* = 0.018, Cohen’s d 1.14; lv-PPA vs HC *p* < 0.001, Cohen’s d 1.35); however, no differences were found between lv-PPA and nfv-PPA patients (*p* = 1.000) (Fig. 1B).

Then, we explored differences in single emotion recognition correcting for age at empathy assessment (Fig. 1A). The three PPA variants had low scores in fear recognition, with no evident differences among them, but not significantly lower compared to HC (*F* [3, 75] = 1.29, *p* = 0.238, partial η^2^ 0.05). Sv-PPA presented lower scores than HC in recognition of disgust (4.00 ± 3.55 vs 7.82 ± 1.81, *p* < 0.001, Cohen’s d 1.68), happiness (8.10 ± 1.91 vs 9.88 ± 0.53, *p* = 0.014, Cohen’s d 1.13), surprise (4.50 ± 3.06 vs 9.00 ± 1.53, *p* < 0.001, Cohen’s d 2.20), sadness (4.10 ± 2.88 vs 7.85 ± 1.77, *p* < 0.001, Cohen’s d 1.70), and anger (3.80 ± 1.31 vs 7.38 ± 1.93, *p* < 0.001, Cohen’s d 1.99). Lv-PPA patients showed a worse performance than HC in recognition of disgust (5.96 ± 2.26 vs 7.82 ± 1.81, *p* = 0.013, Cohen’s d 0.78), happiness (7.96 ± 1.93 vs 9.88 ± 0.53, *p* < 0.001, Cohen’s d 1.13), surprise (6.29 ± 2.25 vs 9.00 ± 1.53, *p* < 0.001, Cohen’s d 1.19), sadness (5.77 ± 2.45 vs 7.85 ± 1.77, *p* = 0.021, Cohen’s d 0.79), and anger (5.22 ± 1.82 vs 7.38 ± 1.93, *p* < 0.001, Cohen’s d 1.09) too. On the other hand, nfv-PPA patients presented worse performance than HC exclusively in recognition of anger (4.55 ± 1.94 vs 7.38 ± 1.93, *p* = 0.002, Cohen’s d 1.42).

Moreover, no significant differences were found among the three PPA variants in single emotion recognition scores, except for a worse performance in surprise recognition in sv-PPA than in nfv-PPA (4.50 ± 3.06 vs 6.88 ± 2.26, *p* = 0.035, Cohen’s d 1.33). Similarly, sv-PPA patients had lower scores in surprise recognition than lv-PPA, but this difference did not reach statistical significance (6.29 ± 2.25, *p* = 0.057, Cohen’s d −1.00).

**Fig. 2:**
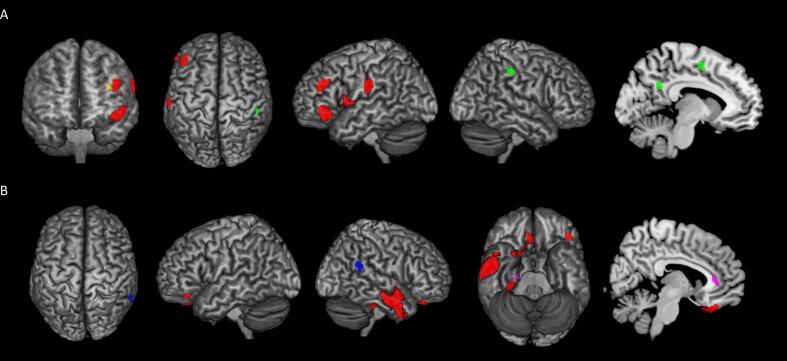
a) Clusters of significant positive correlation between emotion recognition and cerebral metabolic activity in logopenic primary progressive aphasia; b) clusters of significant positive correlation between emotion recognition and cerebral metabolic activity in semantic Primary Progressive Aphasia. Anger: red. Disgust: green. Surprise: yellow. Sadness: blue. Fear: purple. (For interpretation of the references to colour in this figure legend, the reader is referred to the web version of this article.)

Furthermore, we classified patients’ performance in recognizing individual emotions as normal or pathological, and subsequently evaluated whether the percentage of patients with pathological performance differed among HC and the three PPA groups (Fig. 2C).

Concerning sadness recognition, all PPA patients presented a pathological performance more frequently (nfv-PPA 22.22 %, sv-PPA 50 %, lv-PPA 14.81 %) than HC (0 %) (respectively, nfv-PPA vs HC χ^2^ = 7.921, *p* = 0.040, Cramer’s V 0.288; sv-PPA vs HC χ^2^ = 19.17, *p* < 0.001, Cramer’s V 0.660; lv-PPA vs HC χ^2^ = 5.39, *p* = 0.034, Cramer’s V 0.297). Moreover, sv-PPA presented a pathologic sadness recognition more frequently than lv-PPA patients (50 % vs 14.81 %, χ^2^ = 4.908, *p* = 0.041, Cramer’s V 0.364).

Similarly, all PPA patients presented a pathological performance in anger recognition more frequently (nfv-PPA 44.44 %, sv-PPA 60 %, lv-PPA 44.44 %) than HC (5.88 %) (respectively, nfv-PPA vs HC χ^2^ = 8.81, *p* = 0.012, Cramer’s V 0.453; sv-PPA vs HC χ^2^ = 15.21, *p* < 0.001, Cramer’s V 0.588; lv-PPA vs HC χ^2^ = 12.65, *p* < 0.001, Cramer’s V 0.455). No differences in the frequency of pathologic anger recognition were found among the three PPA variants.

As regards happiness recognition, pathologic scores were found in 48.14 % of lv-PPA and 40 % of sv-PPA, with higher frequency than in HC (2.94 %) (respectively, sv-PPA vs HC χ^2^ = 10.53, *p* = 0.007, Cramer’s V 0.489; lv-PPA vs HC χ^2^ = 17.39, *p* < 0.001, Cramer’s V 0.534). The percentage of patients with pathologic performance did not differ between HC and nfv-PPA nor among the three PPA subgroups.

Concerning surprise, a pathologic recognition was detected more frequently in sv-PPA (70 %) and in lv-PPA (33.33 %) than in HC (5.88 %) (sv-PPA vs HC χ^2^ = 19.52, *p* < 0.001, Cramer’s V 0.666; lv-PPA vs HC χ^2^ = 7.67, *p =* 0.008, Cramer’s V 0.353).

Sv-PPA but not nfv-PPA and lv-PPA patients showed pathologic scores in disgust recognition with a higher frequency than HC (60 % vs 2.94 %, χ^2^ = 18.80, *p* < 0.001, Cramer’s V 0.654). The percentage of patients with pathologic performance did not differ between HC and lv-PPA, HC and nfv-PPA nor among the three PPA subgroups. Moreover, sv-PPA presented a pathologic disgust recognition more frequently than lv-PPA patients (60 % vs 14.81 %, χ^2^ = 7.55, *p* = 0.012, Cramer’s V 0.452).

Finally, no differences in the frequency of pathologic performance in fear recognition were found neither between HC and PPA patients nor among the three PPA variants subgroups.

### 3.4 SPM analysis results

The multiple regression model did not show significant correlations between brain metabolic activity and EK-60F total score in any of the three PPA variants. However, significant correlations between single emotions recognition at the EK-60F Test and brain metabolism in both lv-PPA (Table 3, Fig. 2A) and sv-PPA (Table 3, Fig. 2B) were detected. With regards to lv-PPA, significant correlations were found between metabolic activity and recognition of disgust, anger and surprise. In particular, disgust recognition was positively correlated with metabolic activity of right supramarginal gyrus (SMG), left precuneus, left supplementary motor area and right middle cingulate gyrus. On the other hand, anger recognition was positively correlated with metabolism of left inferior frontal gyrus (IFG), left SMG, left middle frontal gyrus (MFG) and left insula. Finally, surprise recognition was positively correlated with metabolic activity in left IFG (*p* < 0.001). No significant correlations between happiness, fear, sadness recognition and brain metabolism were detected in this variant.

**Table 3:** Correlations between emotion recognition and cerebral hypometabolism in in logopenic Primary Progressive Aphasia and semantic Primary Progressive Aphasia at 18F-FDG-PET SPM analysis.

lv-PPA	Cluster Extent (voxels)	MNI coordinates (mm)			T values	*P (FWE-SVC)*
**Disgust**		*x*	*y*	*z*		
Right supramarginal gyrus	*68*	*46*	*−28*	*44*	*4.93*	*0.001*
Left precuneus	*43*	*−14*	*−54*	*30*	*4.86*	*0.002*
Left supplementary motor area	*63*	*−12*	*−2*	*52*	*4.84*	*0.002*
Right middle cingulate gyrus	*49*	*12*	*18*	*36*	*4.73*	*0.002*
**Anger**		*x*	*y*	*z*		
Left inferior frontal gyrus	*124*	*−46*	*30*	*−10*	*5.22*	*0.001*
Left supramarginal gyrus	*75*	*−66*	*−20*	*24*	*4.96*	*0.001*
Left middle frontal gyrus	*56*	*−44*	*36*	*28*	*4.54*	*0.003*
Left insula	*91*	*−50*	*6*	*4*	*4.53*	*0.003*
**Surprise**		*x*	*y*	*z*		
Left inferior frontal gyrus	*38*	*−34*	*22*	*24*	*4.22*	*0.006*

**sv-PPA**	**Cluster Extent (voxels)**	**MNI coordinates (mm)**			**T values**	***P (FWE-SVC)***
**Sadness**		*x*	*y*	*z*		
Right supramarginal gyrus	*30*	*62*	*−48*	*24*	*8.79*	*0.006*
**Anger**		*x*	*y*	*z*		
Right middle temporal gyrus	*318*	*56*	*−2*	*−20*	*11.50*	*0.003*
Right orbitofrontal gyrus	*25*	*20*	*12*	*−28*	*11.27*	*0.002*
Left orbitofrontal gyrus	*16*	*−46*	*36*	*−18*	*10.86*	*0.002*
Right fusiform gyrus	*34*	*30*	*−30*	*26*	*9.86*	*0.008*
Right inferior temporal gyrus	*18*	*46*	*10*	*−48*	*6.63*	*0.03*
**Fear**		*x*	*y*	*z*		
Right anterior cingulate cortex	*24*	*4*	*38*	*2*	*15.00*	*0.001*
Right parahippocampal gyrus	*17*	*24*	*−16*	*−34*	*13.74*	*0.001*

In sv-PPA group, significant correlations were found between brain metabolism and sadness, anger and fear recognition. In particular, sadness recognition was positively correlated with metabolism of right SMG (*p* < 0.001), while anger recognition with metabolism of right middle (MTG) and inferior temporal gyri (ITG), bilateral orbitofrontal gyri and right fusiform gyrus. As for fear recognition, it was positively correlated with metabolic activity in right anterior cingulate cortex (ACC) and right *para*-hippocampal gyrus. No significant correlation was found between disgust, happiness, surprise recognition and brain metabolism in this variant. Finally, no significant correlations between metabolic activity and EK-60F scores were detected in the nfv-PPA variant.

## 4 Discussion

This study explored empathy and emotion recognition deficits across the three main PPA variants (sv-PPA, lv-PPA and nfv-PPA), aiming to differentiate them on the basis of social cognition impairments and to investigate the neural correlates of such impairments.

First, we explored empathy capacity through the IRI questionnaire analysing the changes of such scores from before to after the onset of cognitive symptoms. Interestingly, we detected a significant decrease of Perspective Taking and an increase of Personal Distress scores in all PPA variants. Perspective Taking is generally considered as the main indicator of cognitive empathy (Davis, 1983, Shamay-Tsoory et al., 2009) and other studies have already observed its reduction in sv-PPA (Rankin et al., 2006, Binney et al., 2016, Rankin et al., 2005), nfv-PPA (Hazelton et al., 2017) and lv-PPA (Hazelton et al., 2017, Giacomucci et al., 2023). Therefore, our result confirms the presence of a cognitive empathy deficit, which evolves with the disease’s progression, in all PPA variants. On the other hand, Personal Distress is a measure of emotional contagion (Sturm et al., 2013) and its increase could be secondary to the loss of inhibitory control on this primitive empathic function. Heightened emotional contagion has already been observed in all PPA variants (Hazelton et al., 2017, Giacomucci et al., 2023, Rankin et al., 2005).

Only lv-PPA patients showed a reduction of Fantasy scores from before to after the onset of cognitive symptoms. Together with Perspective Taking, Fantasy subscale explores cognitive empathy (Shamay-Tsoory et al., 2009), but very few studies have used it to evaluate empathy in PPA patients, because its validity is discussed and Perspective Taking subscale is generally preferred (Davis, 1983, Rankin et al., 2006). However, a reduction of Fantasy scores in lv-PPA has already been observed (Hazelton et al., 2017) and it could be a consequence of global cognitive impairment. Indeed, it has been proposed that empathy deficits in lv-PPA might be secondary to the extensive decline of other cognitive functions (Hazelton et al., 2017, Giacomucci et al., 2023).

It is noteworthy that, in the present study, sv-PPA patients did not show impairments in the Fantasy subscale, contrary to results from previous studies (Rankin et al., 2005, Calabria et al., 2009, Henry et al., 2014), which are however scarce and based on a limited number of patients. To discuss this result, it might be suggested that Perspective Taking and Fantasy scores explore two aspects of cognitive empathy that are mediated by different neural pathways, therefore leading to Fantasy preservation in sv-PPA.

Finally, no changes in Empathic Concern scores (a measure of affective empathy) were detected among PPA patients. This result seems to point to a preservation of affective empathy in all PPA variants, with a trend of reduction in sv-PPA. This result is in line with other studies as concerns lv-PPA (Giacomucci et al., 2023) and nfv-PPA (Hazelton et al., 2017). As for sv-PPA, evidence is less consistent, with previous studies showing both a sparing (Binney et al., 2016, Calabria et al., 2009) or a reduction (Rankin et al., 2006, Rankin et al., 2005, Irish et al., 2014) of Empathic Concern. However, it must be noted that in our sample of sv-PPA patients’ temporal atrophy was always left-sided. Indeed, current research has led to the hypothesis that, in sv-PPA, the preservation of affective empathy (and therefore Empathic Concern scores) might depend on the lateralisation of temporal atrophy, with a reduction of empathic concern observed only in right sv-PPA patients (Rankin et al., 2006, Calabria et al., 2009, Irish et al., 2013).

Then, we analysed emotion recognition capacity through the EK-60F Test and found impairments in all PPA variants. In particular, a gradient of sorts emerged, with sv-PPA being the most impaired variant, followed by lv-PPA and, finally, by nfv-PPA. Indeed, sv-PPA patients had lower EK-60F total scores than lv-PPA, nfv-PPA and HC; lv-PPA and nfv-PPA showed lower EK-60F total scores than HC, while no differences were found among the two PPA variants.

Concerning single emotions’ recognition, sv-PPA and lv-PPA showed, compared to HC, impairments in the recognition of all emotions except for fear, while nfv-PPA was impaired only in the recognition of anger. Moreover, sv-PPA had a worse deficit than nfv-PPA in the recognition of surprise. When analysing the percentage of patients with pathological performances in single emotions’ recognition, a similar pattern emerged, highlighting the presence of a severity gradient. In fact, all PPA patients performed worse than HC in sadness and anger recognition, only sv-PPA and lv-PPA patients had a higher percentage of pathological scores than HC in the recognition of happiness and surprise and, finally, only sv-PPA patients had a worse performance than HC in disgust recognition. Moreover, sv-PPA had a higher percentage of pathological scores in disgust and sadness recognition as compared to lv-PPA.

As regards sv-PPA, these findings are in line with previous studies showing an extensive impairment of emotion recognition, which is described as primary and independent of other cognitive disturbances (Fittipaldi et al., 2019, Kumfor et al., 2013, Irish et al., 2013). In lv-PPA, emotion recognition capacity has generally been referred to as mostly preserved (Piguet et al., 2015) or only slightly impaired (Hazelton et al., 2017, Giacomucci et al., 2023). Our study confirms the latter hypothesis, with the presence of a deficit in the recognition of all emotions (except for fear), although milder than that of sv-PPA patients. Contrary to our results, previous research points to a more severe impairment in nfv-PPA as compared to lv-PPA (Fittipaldi et al., 2019, Piguet et al., 2015). Nevertheless, our findings are in line with those same studies in observing that, in nfv-PPA patients, deficits in emotion recognition specifically involve anger and sadness. The preservation of disgust recognition has already been observed in this variant and it has been correlated with the sparing of anterior insula (Kumfor et al., 2013).

Moreover, it is interesting to discuss the preservation of fear recognition in PPA variants. In fact, no differences in the recognition of this emotion have been observed between PPA groups and HC nor among PPA variants. However, it must be noted that this is not a result of a better recognition of fear by PPA patients, but it is a consequence of a scarce capacity of recognition shown by both PPA patients and HC. Indeed, among all emotions, fear is the most difficult to identify even for healthy individuals, so that the cut-off value for pathological performance in the recognition of this emotion is lower than that of other emotions (Dodich et al., 2014). However, further research with larger groups of healthy controls might help to identify possible differences between HC and PPA patients that were not detected in our study.

Lastly, we explored the correlations between EK-60F Test scores and metabolic activity on [18F]FDG-PET, aiming to identify specific metabolic patterns associated with emotion recognition deficits in PPA variants. Our results showed significant correlations only in sv-PPA and lv-PPA. In particular, as concerns sv-PPA, we found positive correlations between anger and metabolic activity of right inferior temporal gyrus, middle temporal gyrus, fusiform area and bilateral orbitofrontal cortex; between fear and right anterior cingulate cortex and right *para*-hippocampal gyrus’ metabolism; and between sadness and right supramarginal gyrus’ metabolic activity. These findings confirm an involvement of temporo-insular structures in emotion recognition (particularly anger), which is consistent with results from previous studies (Fittipaldi et al., 2019, Kumfor et al., 2013). Indeed, temporal cortices have a fundamental role in the processing of various socioemotional stimuli (Sturm et al., 2013). Moreover, the fusiform area is involved in face recognition (Rankin et al., 2006) and the anterior cingulate cortex is a core structure of the emotion recognition network, together with anterior insula. Its involvement in empathy for pain (Bernhardt and Singer, 2012, Lamm et al., 2011) and in negative emotions’ recognition has been extensively described (Kumfor et al., 2013, Bernhardt and Singer, 2012, Wicker et al., 2003). The orbitofrontal cortex is part of the cognitive empathy network (with a regulatory function) and it is known to play a role in the recognition of emotions (Rankin et al., 2006, Bernhardt and Singer, 2012, Bartochowski et al., 2018). A role of the supramarginal gyrus in emotion recognition has also been described (Wada et al., 2021), as this area is a component of the inferior parietal lobule, which is involved in numerous empathic functions and, in particular, in self-other distinction (Decety and Jackson, 2004). The involvement of predominantly right structures supports the hypothesis of a dissociation between left and right temporal cortices in sv-PPA (Rankin et al., 2006, Wong and Gallate, 2012), where atrophy of right temporal structures leads to social cognition deficits independently of language disruption, which is regulated by left temporal areas.

On the other hand, in lv-PPA group, we found positive correlations between anger recognition and metabolic activity of right supramarginal gyrus, left precuneus, left supplementary motor area and right middle cingulate gyrus; between disgust and metabolism of left inferior frontal gyrus, middle frontal gyrus, left supramarginal gyrus and left insula; and between surprise and left inferior frontal gyrus metabolic activity. These results are consistent with the well-known role of the insulo-cingulate network in emotion recognition, especially of negative emotions (Kumfor et al., 2013, Bernhardt and Singer, 2012, Wicker et al., 2003). Moreover, they highlight an involvement of the inferior frontal gyrus, which takes part in the affective empathy network (Bartochowski et al., 2018) and whose role in emotion recognition has already been described by other studies (Bernhardt and Singer, 2012, Wada et al., 2021). Finally, the lack of significant correlations found in nfv-PPA can be explained by the small sample size of this group.

Our study presents some limitations. First, the exploratory nature of the study, thus findings should be interpreted with caution and require confirmation in larger, independent cohorts. Second, the use of the IRI: despite being an extensively used test in studies exploring empathy, its reliability is discussed, particularly when it is administered only to the caregivers (Shany-Ur et al., 2012). However, informants’ scores can capture real-life empathy capacity independently from the patients’ anosognosia (Fittipaldi et al., 2019), thereby proving advantageous in this type of disease. Moreover, the IRI does not provide a comprehensive evaluation of empathy, as it explores a limited number of aspects. In the future, the use of complimentary tests could be useful to better investigate other components of empathy and social cognition. Furthermore, although a consistent number of patients was included in the study, a larger sample size, particularly of the least represented groups (sv-PPA and nfv-PPA), could undoubtedly contribute to a more comprehensive understanding of empathy deficits associated with this syndrome. In particular, an enlargement of nfv-PPA group could allow to obtain significant results as concerns the neural correlates of emotion recognition in this variant. Furthermore, for SPM analyses, we were not able to use the Total Grey Matter Volume as further covariate to correct for cortical atrophy.

On the other hand, this study has some remarkable strengths. It is one of the most recent studies investigating empathy and emotion recognition in all three PPA variants, aiming to compare them to healthy controls and to highlight specific differences which could help in early-stages diagnostic process. Both empathy and emotion recognition deficits were explored through the IRI and EK-60F Test respectively. In order to gain a more comprehensive understanding of the emotion recognition capacity, EK-60F scores were analysed in two ways: one was based on raw scores of the single subscales, while the other one was based on cut-offs for pathological performances and, therefore, on the percentage of patients with a pathological performance in single emotions’ recognition. This approach allowed for the acquisition of a more detailed and multifaceted perspective on the emotion recognition deficits exhibited by PPA patients. Moreover, it is also one of the few studies to explore the neural correlates of social cognition deficits in PPA through the use of SPM analysis of FDG-PET.

In conclusion, our results support the limited existing literature, demonstrating impaired empathy and emotion recognition in PPA, with varying degrees of impairment across the three variants. In particular, we demonstrated a severe impairment of empathy and emotion recognition in sv-PPA patients, with a reduction of cognitive empathy, an increase of emotional contagion and deficits in the recognition of all emotions, in line with previous research (Fittipaldi et al., 2019). It must be kept in mind that these patients share several similarities with bv-FTD (Van Langenhove et al., 2016), in which the presence of an empathy deficit is indeed one of the core diagnostic features (Bartochowski et al., 2018, Dermody et al., 2016). Interestingly, affective empathy seems to be, at least in part, preserved, probably due to the left lateralisation of temporal atrophy. Lv-PPA shows similar deficits both in empathy and in emotion recognition, but they are milder than those observed in sv-PPA. A reduction of FT score in this variant suggests that the cognitive empathy impairment might be secondary to global cognitive disruption. Finally, nfv-PPA is the less impaired variant, especially in emotion recognition, as patients exhibit deficits exclusively in the recognition of anger and sadness. The relative sparing of social cognition functions in this variant could be explained by recent theories, which describe nfv-PPA as a motor syndrome that reveals itself through language (Ruksenaite et al., 2021), in particular with motor speech impairments (Gorno-Tempini et al., 2011). Therefore, because of its motor nature, cognitive functions as a whole could be relatively spared in nfv-PPA variant. Moreover, the evaluation of neural correlates has confirmed the involvement of structures typically known to have a role in the emotion recognition network, such as the insulo-cingulate circuit, temporal and orbitofrontal cortices.

Overall, our findings confirm that PPA variants are characterized not only by language impairments, but also by other cognitive deficits, particularly in social cognition. This characterization may prove to be useful for a better and more comprehensive understanding of the complexity of PPA syndrome.

## CRediT authorship contribution statement

**Giulia Giacomucci:** Writing – review & editing, Writing – original draft, Visualization, Validation, Supervision, Methodology, Investigation, Formal analysis, Data curation, Conceptualization. **Alice Pieri:** Writing – review & editing, Writing – original draft, Validation, Supervision, Methodology, Investigation, Formal analysis, Data curation. **Valentina Moschini:** Validation, Supervision, Methodology, Investigation, Formal analysis, Data curation. **Chiara Crucitti:** Visualization, Validation, Data curation. **Sonia Padiglioni:** Visualization, Validation, Data curation. **Carmen Morinelli:** Visualization, Validation, Data curation. **Giulia Galdo:** Visualization, Validation, Investigation, Data curation. **Filippo Emiliani:** Visualization, Validation, Data curation. **Matilde Nerattini:** Visualizaion, Validation, Resources, Data Curation, Investigation. **Silvia Bagnoli:** Visualization, Validation, Data curation. **Assunta Ingannato:** Visualization, Validation, Data curation. **Sandro Sorbi:** Visualization, Validation, Supervision. **Benedetta Nacmias:** Visualization, Validation, Supervision. **Valentina Berti:** Writing – review & editing, Visualization, Validation, Supervision, Resources, Project administration, Funding acquisition. **Valentina Bessi:** Writing – review & editing, Visualization, Validation, Supervision, Resources, Project administration, Funding acquisition.

## Funding

This research project was funded by Tuscany Region (project code D19G22000640002), and by the Ministry of University and Research (MUR), National Recovery and Resilience Plan (NRRP), with the project MNESYS (PE0000006) – A Multiscale integrated approach to the study of the nervous system in health and disease (DN. 1553 11.10.2022) and with the Projects of Relevant National Interest (PRIN-2022WK7NHC)

## Declaration of competing interest

The authors declare that they have no known competing financial interests or personal relationships that could have appeared to influence the work reported in this paper.

## Data Availability

Data will be made available on request.
