# Supplementary material for: Beyond language: empathy and emotion recognition deficits in primary progressive aphasias
Source: Neuroimage Clin. 2025 Jul 23;48:103852. doi: 10.1016/j.nicl.2025.103852 (PMC12337676; doi:10.1016/j.nicl.2025.103852)
Supplement: Supplementary Data 1 [file mmc1.docx]

**Supplementary Materials**

**Supplementary Table 1. Comparison of CSF biomarkers and amyloid-PET positivity among non-fluent, semantic and logopenic Primary Progressive Aphasias (nfv-PPA, sv-PPA, lv-PPA)**

|  | **nfv-PPA** | **sv-PPA** | **lv-PPA** | ***p*** | ***p* between nfv-PPA and sv-PPA** | ***p* between nfv-PPA and lv-PPA** | ***p* between sv-PPA and lv-PPA** |
| --- | --- | --- | --- | --- | --- | --- | --- |
| CSF Aβ1-42 | 949.42±545.73 | 1101.90±448.22 | 477.31±160.68 | **<0.001** | 0.898 | **<0.001** | **<0.001** |
| CSF Aβ1-42/1-40 ratio | 0.081±0.030 | 0.095±0.020 | 0.048±0.012 | **<0.001** | 0.306 | **<0.001** | **<0.001** |
| CSF t-tau | 520.68±178.50 | 375.40±96.15 | 767.15±409.01 | **0.004** | 0.935 | 0.099 | **0.006** |
| CSF p-tau | 64.30±35.65 | 43.48±14.01 | 127.62±79.55 | **<0.001** | 1.000 | **0.016** | **0.002** |
| Amy-PET positivity | 0/1 (0%) | 2/4 (50%) | 12/12 (100%) | - | 1.000 | 0.077 | 0.050 |

Values are reported as mean and standard deviation, or as number and percentage. Statistically significantly different values between the groups are reported as **bold character**. Statistical significancy at *p*<0.05

**Supplementary Table 2. Comparison of facial emotion recognition ability in non-fluent, semantic and logopenic Primary Progressive Aphasia (nfv-PPA, sv-PPA, lv-PPA) and healthy controls**

|  | **HC** | **nfv-PPA** | **sv-PPA** | **lv-PPA** | **F** | ***p*** | **par. η^2^** | ***p* between HC and nfv-PPA** | ***p* between HC and sv-PPA** | ***p* between HC and  lv-PPA** | ***p* between nfv-PPA and sv-PPA** | ***p* between nfv-PPA and lv-PPA** | ***p* between sv-PPA and lv-PPA** |
| --- | --- | --- | --- | --- | --- | --- | --- | --- | --- | --- | --- | --- | --- |
| **EK-60 F total score** | 48.58±6.03 | 39.89±9.34 | 29.58±11.98 | 38.37±6.67 | ***19.83*** | ***<0.001*** | *0.43* | ***0.018*** | ***<0.001*** | ***<0.001*** | ***0.024*** | *1.000* | ***0.015*** |
| **Execution time (sec)** | 244.44±48.48 | 415.66±82.56 | 443.30±146.23 | 442.87±114.59 | ***23.23*** | ***<0.001*** | *0.50* | ***<0.001*** | ***<0.001*** | ***<0.001*** | *1.000* | *1.000* | *1.000* |
| **Anger** | 7.38±1.93 | 4.55±1.94 | 3.80±1.31 | 5.22±1.82 | ***13.72*** | ***<0.001*** | *0.35* | ***0.002*** | ***<0.001*** | ***<0.001*** | *1.000* | *1.000* | *0.113* |
| **Disgust** | 7.82±1.81 | 5.44±2.06 | 4.00±3.55 | 5.96±2.26 | ***8.71*** | ***<0.001*** | *0.26* | *0.069* | ***<0.001*** | ***0.026*** | *0.848* | *1.000* | *0.109* |
| **Fear** | 4.20±2.87 | 3.11±3.18 | 2.50±2.22 | 3.33±2.33 | *1.29* | *0.238* | *0.05* | *1.000* | *0.487* | *1.000* | *1.000* | *1.000* | *1.000* |
| **Happiness** | 9.88± 0.53 | 8.88±2.61 | 8.10±1.91 | 7.96±1.93 | ***7.40*** | ***<0.001*** | *0.23* | *1.000* | ***0.014*** | ***<0.001*** | *1.000* | *0.714* | *1.000* |
| **Sadness** | 7.85±1.77 | 5.88±2.84 | 4.10±2.88 | 5.77±2.45 | ***8.43*** | ***<0.001*** | *0.25* | *0.425* | ***<0.001*** | ***0.021*** | *0.217* | *1.000* | *0.114* |
| **Surprise** | 9.00±1.53 | 6.88±2.26 | 4.50±3.06 | 6.29±2.25 | ***15.14*** | ***<0.001*** | *0.38* | *0.154* | ***<0.001*** | ***<0.001*** | ***0.035*** | *1.000* | *0.057* |

Values are reported as mean and standard deviation. Differences in IRI subscales and in single emotion recognition scores are adjusted for age at empathy evaluation. EK-60 F total score is corrected for age and years of education. Statistically significantly different values between the groups are reported as **bold character**. Statistical significancy at *p*<0.05
